# Investigation of timing of surgery and other factors possibly influencing outcome in dogs with acute thoracolumbar disc extrusion: a retrospective study of 1501 cases

**DOI:** 10.1186/s13028-021-00596-w

**Published:** 2021-08-23

**Authors:** Almut Immekeppel, Stefan Rupp, Stanislas Demierre, Kai Rentmeister, Andrea Meyer-Lindenberg, Julia Goessmann, Monty Siddartha Bali, Fenella Schmidli-Davies, Franck Forterre

**Affiliations:** 1 Department of Clinical Veterinary Science, Vetsuisse-Faculty, Small Animal Clinic, University of Bern, Bern, Switzerland; 2 Tierklinik Hofheim, Small Animal Clinic, Hofheim am Taunus, Germany; 3 Neurologie Veterinaire, Small Animal Practice, Ecublens, Switzerland; 4 Tieraerztliche Praxis für Neurologie, Small Animal Practice, Dettelbach, Germany; 5 Department of Clinical Veterinary Sciences, Small Animal Clinic for Surgery and Gynecology, Ludwig-Maximilian-University-Munich, Munich, Germany; 6 Tierklinik Stommeln, Nettegasse 122, 50259 Pulheim, Germany

**Keywords:** Acute deterioration, Grade of severity, Intervertebral disc extrusion, Loss of deep pain perception, Physical rehabilitation, Time of recovery

## Abstract

**Background:**

Intervertebral disc extrusions in the thoracolumbar region are a common spinal neurologic disorder in dogs and usually considered a neurological emergency. Several factors, like timing of surgery, have previously been analysed in order to determine the effect on outcome and time of recovery. Most studies have investigated one defined population of dogs and the influence of a single factor on the overall outcome. In this retrospective study, a large cohort of dogs and the influence of one or combinations of several factors on outcome and time of recovery were analysed.

**Results:**

The bivariate analysis demonstrated a significant association between the following variables and the time of recovery: the time span between the onset of clinical signs and surgery (Cramers Phi $\varphi^{\prime}$ = 0.14; P = 0.003), the grade of severity ($\varphi^{\prime}$ = 0.23; P < 0.001) and the implementation of physical rehabilitation ($\varphi^{\prime}$ = 0.2; P < 0.001). However, the analysis of a multivariable regression model demonstrated that a significant correlation only exists between the time span between the onset of clinical signs and surgery and the overall outcome (P = 0.007), as well as between the grade of severity and the time of recovery (P < 0.001). The percentage of dogs with lacking deep pain perception (DPP) that had to be euthanised due to their neurological condition, decreased from 20.0 to 2.9% when physical rehabilitation was implemented. Additionally, the proportion of dogs (same group) that improved to reach an ambulatory status increased from 80.0 to 91.4%.

**Conclusion:**

The results of the bivariate analysis demonstrated several correlations between some variables and overall outcome or time of recovery, whereas the multivariable regression model demonstrated only two associations. The time span between the onset of clinical signs and surgery was significantly associated with the overall outcome. We therefore suggest that a surgical intervention should be performed without unreasonable delay. Due to the correlation between the grade of severity and time of recovery, owners of dogs with more severe neurological deficits prior to surgery should be informed about the presumably prolonged time of recovery.

## Background

Intervertebral disc extrusions in the thoracolumbar region are a common spinal neurologic disorder in dogs. Multiple studies have been conducted in order to determine whether some breeds have a higher incidence of intervertebral disc disease and if they are more prone to have better or worse outcomes [1–3]. Among chondrodystrophic breeds, severity and progress of intervertebral disc degeneration can vary greatly and therefore lead to various clinical presentations [4]. Many studies have focused on determining factors that influence the progression of the disease and the clinical outcome of patients. It is generally recognized that loss of deep pain sensation is a strong clinical prognostic indicator and is commonly associated with a guarded to poor prognosis concerning locomotory outcome [1, 3, 5–11]. One study defined the presence of postoperative voluntary motor function as the most important prognostic factor [12]. Peracute onset and progression of clinical signs have also been postulated to have a strong influence on the clinical outcome. Scott [10] stated that a peracute onset of clinical signs was associated with a worse outcome, whereas Olby et al. and Ferreira et al. [1, 2] were not able to demonstrate an association between a peracute onset and a poorer outcome. Therefore, it remains inconclusive whether the onset of clinical signs can be used as a reliable prognostic factor. A further key discussion point is the potential influence of the time span from onset of clinical signs until surgery on the outcome and the time of recovery. The majority of studies so far have focused on dogs that had lost their deep pain perception (DPP) and evaluated to what extent prompt surgical intervention affected the outcome. Scott and Duval [3, 10] stated that in case of loss of DPP, an early surgical intervention (during the first 12–48 h) was recommended. Duval et al. [3] moderated this statement by adding that an accurate time frame for the given recommendation still needs to be proven. In a study population of paraplegic dogs, Ferreira et al. [2] were unable to detect an association between outcome and duration of clinical signs prior to surgery. Due to these contradictory findings, further studies with larger cohorts are warranted to allow significant conclusions.

The aim of our study was therefore to investigate possible prognostic factors for outcome in dogs with thoracolumbar intervertebral disc extrusions in a large dog population. In light of the current literature on thoracolumbar intervertebral disc extrusions, our main hypotheses were that the outcome would be negatively affected by a longer time span before surgery and a more severe neurologic grade. We suspected that a short time span between first clinical signs and surgery would lead to shorter recovery times and that animals with an acute onset would have a worse clinical outcome. Additionally, the possible interaction of different variables (duration, grade of severity, progression and physical rehabilitation) in relation to outcome and time of recovery was investigated. A further interest was to evaluate the role of physical rehabilitation and to estimate to what degree it can improve clinical outcome and shorten time of recovery.

We differentiated between general factors (breed, age, gender, progression, severity of clinical signs), therapeutic factors (surgical treatment, premedication, physical rehabilitation) and focused specifically on the effect of timing of surgery.

## Methods

In this multicentered, retrospective study medical records between 2007 and 2012 from six referral centers were reviewed for dogs with acute thoracolumbar intervertebral disc extrusion [Hansen type 1 confirmed via computed tomography (CT) or magnetic resonance imaging (MRI)], which underwent surgical decompression. In order to be included in the study, the following information had to be available: (1) patient signalment: age, gender, weight, breed, (2) clinical signs: date of onset of clinical neurological signs (including the presence of pain), severity of neurological deficits (including the presence of pain), localization of disc extrusion, time between onset of clinical signs and surgery, progression of clinical signs, (3) therapy: surgical treatment, premedication, physical rehabilitation and clinical follow-up. The severity of neurologic deficits was assessed and graded [1–5] at the time the dog was presented to the clinic. A change (if present) in the neurologic status between the time of onset of clinical signs and the time of surgery had to be clearly documented including the timepoint and characterization of the observed change. Patients suffering serious cardiovascular complications or other acute adverse events during surgery, that potentially affected outcome, were excluded from the study. We defined a successful outcome as regaining a normal ambulatory status. The definition “unsuccessful outcome” included the following outcomes: ambulatory with mild proprioceptive deficits, exacerbated neurologic deficits in comparison to the presurgical status, recurrence of clinical signs and euthanasia. When dogs did not receive their post-surgical treatment at the referral clinic, a status from the referring veterinarian was given approximately 4 weeks after surgery and was included in the dog’s medical record. This information was further updated when the referring veterinarian had seen the dog for a final check-up. The final recheck was defined as the moment after which no further progress was expected and the neurological condition was considered stable. If this information was not available from the referral clinic, the owner was contacted and this information retrieved (n = 20). All medical records were reviewed by a single veterinarian (AI). A scoring chart to compare the available data with the prerequisites of the study design was designed. If the available information did not fullfill the requirements, or if the explanations in the medical records were insufficient or unclear to the reviewer, the respective case was not included in the study.

### Patients

All breeds were recorded, but for statistical analysis of this particular variable only breeds that were represented by more than 50 dogs were considered. Concerning gender distribution, the neutering status was not taken into account. For analysis of the variable ‘age’, dogs were grouped as follows: < 4 years, 4 to < 7 years, 7 to < 10 years and > 10 years. Concerning body weight, patients were grouped into the following categories: < 5 kg; 5.0 to < 10 kg, 10.0 to < 20 kg and > 20 kg.

### Clinical signs

Based on the neurological exam performed by a veterinarian at admission, dogs were allocated to one of five grades: (1) pain only, (2) ambulatory paraparesis, (3) non-ambulatory paraparesis, (4) paraplegia with intact DPP, and (5) paraplegia with loss of DPP). The presence of DPP was assessed by clamping the digits of the hindlimbs with a forceps. The behaviour of the patient, following this noxious stimulus was observed (turning of the head, attempting to bite, growling etc.). The implemented grading system was modified after Aikawa et al. and Levine et al. [4, 13].

The progression of clinical signs was categorized into three groups: (1) acute deterioration (within < 10 h), (2) subacute deterioration (within 10—24 h) or (3) stable. This information was most commonly obtained from the report sent by the referring veterinarian and only in a minority of cases based on the history reported by owners.

The intervertebral disc extrusion was anatomically classified as “thoracic” (T2 to T11), “thoracolumbar” region (between T11 and L2) or “lumbar” (L2 to L7).

### Treatment

The different premedication protocols included no premedication, nonsteroidal anti-inflammatory drugs (NSAID), steroids alone or steroids in combination with NSAID. Protocols could not be consistently recorded from the history. Due to the lack of precise data in this large cohort of patients, the influence of anti-inflammatory premedication before surgery on recovery was not evaluated in the present study.

The time span between onset of clinical signs and surgical procedure was recorded from the patient history. Dogs were classified as having surgery on the day of onset of clinical signs (day 0), 1–2 days, 3–5 days or > 5 days after onset of clinical signs.

For confirmation of the diagnosis, CT or MRI was used, depending on the availability of diagnostic imaging facilities at the different referral centers. A cerebrospinal fluid tap was not routinely performed. Depending on the imaging findings and the preference of the surgeon a hemilaminectomy without fenestration, a hemilaminectomy with a single fenestration or a hemilaminectomy with multiple fenestrations was performed.

### Clinical outcome

Clinical outcome was assessed via follow up examinations either at the referral clinic or by the referring veterinarian. Outcome (final status) was categorized into the following groups: (1) recovery of normal ambulatory status, (2) ambulatory with mild proprioceptive deficits, (3) exacerbated neurologic signs (in comparison to the presurgical status), (4) recurrence of clinical neurological signs, (5) euthanasia (in association with the neurological status) and (6) death unrelated to the neurological condition. For statistical analysis, outcomes were grouped into “successful” (outcome 1) and “not successful” (outcomes 2–5). Animals that died for other reasons than their neurological condition were not considered in the statistical analysis.

In order to adequately assess recovery, the patients were divided into two groups, depending on whether the dog was ambulatory prior to surgery or not. In dogs which were non-ambulatory prior to surgery, the duration of recovery was defined as the time following surgery until the patient regained the ability to walk without assistance. Ambulation was defined as being able to walk a distance of approximately 10 m. In dogs which were ambulatory prior to surgery, the time of return to a normal, pain-free status was assessed. This information was provided by the referring veterinarian. The time from surgery until the final follow up was categorized as < 2 weeks, 2 to < 4 weeks, 4 weeks to < 3 months and > 3 months.

Data on whether physical rehabilitation had been performed was obtained either from the clinic, the referring veterinarian or directly from the owners [n = 5 (0.3%)] if this information was unavailable in the hospital files. Dogs were grouped as having received physical rehabilitation or not. As protocols of physical rehabilitation varied among clinics and practices, dogs that were grouped into having received a physical rehabilitation underwent a minimum of either passive movement, manual therapy, massage or a combination of the aforementioned.

### Statistical analysis

A bivariate analysis was used, in order to investigate a relationship between the two paired data sets (variables). The data was analysed by using the Chi squared test.

Additionally, a multiple regression analysis was used to find correlations between the data sets. As the Phi coefficient can only be used in quadratic tables (equal number of columns and lines), the Cramers-Phi ($\varphi ^{\prime}$) was implemented to calculate the degree of association for rectangular tables in which the number of columns was greater than the number of lines (and vice versa). Due to the nominal character of our data, only Phi and Cramers-V were interpreted: for nominal scaled variables, the correlation varies between the values zero and one. A value of 0 signifies no correlation between the tested variables. The classification of correlation strength according to Cohen [14] was used:

$$ \varphi ^{\prime} = \sqrt {\frac{{\chi^{2} }}{{N\left( {r - 1} \right)}}} = \frac{{\varvec{w}}}{{\sqrt {r - 1} }}, $$

$$ {\varvec{w}} = \varphi^{\prime}\sqrt {r - 1} , $$

Small: **w** = 0.10,

Medium: **w** = 0.30,

Large: **w** = 0.50.

Therefore, $\varphi ^{\prime}$ and Cramer-V were calculated as measures of correlation.

α was set at 0.05. The threshold of a meaningful correlation was set at 0.1. Each parameter was coded into a numeric grading system. The statistical analysis was performed via PSPP 1.2.0, which is distributed by GNU Operating System and sponsored by Free Software Foundation, Inc.

## Results

### Patients

1501 dogs met the inclusion criteria. Twenty-two dogs had to be excluded from statistical evaluation since they died from reasons unrelated to their neurological condition shortly after surgery. Therefore, for statistics the total number of dogs used for calculation was 1479. Eighty-six different breeds were included in the study. The most common breed was the Dachshund (n = 477; 32.3%), followed by mixed breeds (n = 321; 21.7%), French bulldogs (143; 9.7%), Beagles (n = 55; 3.7%), Cocker Spaniels (n = 43; 2.8%), German Shepherd Dogs and Jack Russell Terriers (each n = 35; 2.4%). The remaining breeds each represented less than 2.4% of the study population. 58.5% of dogs were male and 41.5% female. In total, 779 dogs (52.7%) were chondrodystrophic (Dachshund, French Bulldog, Pekingese, Cocker Spaniel, etc.). Analysis of age distribution showed that dogs younger than 4 years constituted 9.9% of the population, dogs between 4 and 7 years constituted 51.7%, dogs between 7 and 10 years constituted 25.9% and dogs older than ten years constituted 12.5%. Dogs with a body weight less than 5 kg constituted 8.6%, dogs between 5 and 10 kg constituted 42.0%, dogs between 10 and 20 kg constituted 35.2% and dogs with a body weight of more than 20 kg constituted 14.3% of the population.

### Clinical signs

On clinical neurological examination, 304 animals (20.6%) were only painful (grade 1), 493 (33.3%) showed an ambulatory paraparesis (grade 2), 446 (30.2%) were non-ambulatory paraparetic (grade 3), 182 (12.3%) were paraplegic with intact deep pain perception (grade 4) and 54 dogs (3.7%) were paraplegic with loss of deep pain perception (grade 5).

Four hundred and seventy-three (32.0%) dogs experienced a worsening of their neurologic state between the onset of clinical signs and surgery, whereas 489 patients (33.1%) remained clinically stable in this time. Usually, changes of the neurologic state occurred during the first 72 h.

Intervertebral disc extrusions were more frequent in the thoracolumbar region [n = 698 (47.2%)] than in the lumbar [n = 489 (33.1%)] or in the thoracic [n = 290 (19.6%)] region of the spine. A concomitant thoracolumbar and lumbar [n = 1 (0.1%)], as well as a concomitant thoracic and thoracolumbar extrusion [n = 1 (0.1%)] were also present.

### Treatment

Surgery was performed on the day of onset of clinical signs (day 0) in 354 (24.0%) animals, between day 1–2 in 453 animals (30.6%), between the third to fifth day in 255 (17.2%) and after > 5 days in 417 animals (28.2%).

In 934 patients (63.2%) a hemilaminectomy without fenestration was performed, in 468 (31.6%) a hemilaminectomy with a single fenestration and in 23 (1.6%) a hemilaminectomy with multiple fenestrations, respectively. In the remaining 54 cases (3.7%) of multifocal disc extrusions, a combination of the mentioned surgical methods was chosen.

### Clinical outcome

Analysis throughout all neurologic grades revealed that after surgical treatment, 89.8% of the population regained completely normal locomotory function. The locomotory function was always assessed by a veterinarian. 5.8% of the population remained ambulatory with mild proprioceptive deficits and 0.7% were non-ambulatory. A recurrence of clinical signs was observed (clinical follow up) in 0.7% and 3.0% of the dogs were euthanized due to their neurologic status. Twenty-two dogs died or were euthanised for reasons unrelated to their neurological condition during the survey period. A necropsy was not performed. These dogs were excluded from statistical evaluation.

Most dogs (90.5%) that were presented to the clinic with pain only regained a normal ambulatory status after surgery while 7.9% were ambulatory but showed mild proprioceptive deficits and 1.3% were euthanised due to complications. Of the grade 2 patients (ambulatory paraparesis), 89.5% returned to a normal ambulatory status, 7.9% were ambulatory with mild proprioceptive deficits and 2.6% were euthanised. 90.8% of grade 3 patients (non ambulatory paraparesis) achieved a normal ambulatory status, in 4.0% mild proprioceptive deficits were present and 2.7% were euthanised. Recovery to a normal ambulatory status was seen in 87.9% of grade 4 patients (paraplegia with intact DPP), an ambulatory status with mild proprioceptive deficits was achieved in 2.7% of cases and 5.5% were euthanised. Of the patients with paraplegia and loss of DPP, 87.0% returned to a normal ambulatory status and 9.3% were euthanised (Table 1).

**Table 1:** Outcome of the 1479 dogs with thoracolumbar intervertebral disc extrusion distributed according to the neurologic grade prior to surgery

Grade of severity prior surgery	Number of cases	Number of cases with successful outcome
Pain only (grade 1)	304	275 (90.5%)
Ambulatory paraparesis (grade 2)	493	441 (89.5%)
Non-ambulatory paraparesis (grade 3)	446	405 (90.8%)
Paraplegia with intact DPP (grade 4)	182	160 (87.9%)
Paraplegia with loss of DPP (grade 5)	54	47 (87.0%)
Total	1479	1328

Of the dogs that were presented to the clinic with pain only (n = 304), 199 (65.5%) recovered within the first 2 weeks, 63 (20.7%) after 3–4 weeks, 28 (9.2%) within 1–3 months and 10 (3.3%) required more than 3 months to recover. In the remaining four cases, there was contradictory information from the referring veterinarian and the clinic.

Less than half (40.7%, n = 22 animals) of dogs that had lost deep pain perception (n = 54) made a full recovery within the first 2 weeks. The distribution among the remaining three groups was 16.7% (n = 9 animals) per group. In the remaining five cases, the information on outcome was inconclusive.

Of the 354 dogs, that underwent surgery on the day of onset of clinical signs (day 0), 326 (92.1%) regained a normal locomotory status postoperatively, nine (2.5%) were ambulatory with mild proprioceptive deficits and one (0.3%) had exacerbated neurologic deficits in comparison to the presurgical status. One dog (0.3%) had recurrence of clinical signs and 17 patients were euthanised (4.8%).

Of the 453 dogs that underwent surgery between day 1 and 2 after onset of clinical signs, 412 (90.9%) regained a normal ambulatory status, 15 (3.3%) were ambulatory with mild proprioceptive deficits, seven (1.5%) had exacerbated neurologic deficits in comparison to the presurgical status, four (0.9%) had recurrence of clinical signs and 15 dogs were euthanised.

If the time span between onset of clinical signs and surgery was 2–5 days, (n = 255) 229 (89.8%) regained normal ambulation, 17 (6.7%) were ambulatory with mild proprioceptive deficits, two (0.8%) had exacerbated neurologic deficits in comparison to the presurgical status, three (1.2%) had a recurrence of clinical signs and four (1.6%) were euthanised.

In cases in which more than 5 days had passed until surgery was performed (n = 417), 361 (86.6%) achieved a normal ambulatory status postoperatively, 45 (10.8%) were ambulatory with mild proprioceptive deficits, one (0.2%) had exacerbated neurologic deficits in comparison to the presurgical status, two (0.5%) had a recurrence of clinical signs and eight (1.9%) were euthanised.

A total of 714 animals (48.3%) did not receive a physical rehabilitation, whereas 690 animals (46.7%) did receive this adjunctive treatment. The greatest difference in outcome was seen in grade 5 patients. The percentage of dogs that had to be euthanised due to their poor neurologic status decreased from 20.0 to 2.9% if physical rehabilitation was implemented and the percentage of dogs that regained a normal ambulatory status increased from 80.0 to 91.4%. (Table 2).

**Table 2:** Outcome of 1479 dogs with thoracolumbar intervertebral disc extrusion distributed according to the implementation of physical rehabilitation

Physical rehabilitation	Number of cases	Number of cases with successful outcome
Yes	690	620 (89.8%)
No	714	641 (89.7%)
Contradictory data	75	67 (89.3%)
Total	1479	1328

### Patients

Within the observed population of dogs, most were between 4 and < 7 years (51.6%) of age. When this group was split into female and male dogs, the observed distribution of male:female differed from the expectation of 50:50. A Chi squared test was performed and a value of 0.02 was estimated. We can therefore state that a significant statistical difference between the observed and the expected distributions exists. Additionally, the most common body weight categorisation was dogs with a body weight between 5.1 and < 10.0 kg (42.0%). By splitting this group into female and male dogs, differences between the observed and the expected distribution were observed. The Chi squared test confirmed that a significant statistical difference between the observed and the expected distribution exists (Chi^2^ < 0.001).

We note that these comparisons are only made within the study population, as it was not possible to compare the distribution in our sample to the expected distribution in the general population of dogs.

### Clinical signs

The bivariate analysis could not find a significant association between the neurological grade and the outcome (P = 0.761), but a significant association between the neurological grade and the recovery time was detected ($\varphi ^{\prime}$ = 0.23; P < 0.001). This finding could be confirmed in a multivariable regression model (P < 0.001). In addition, a crosstable (2 × 2) was calculated exclusively for dogs with severity grade 5 (grade 5: yes or no). Outcome was again grouped into “successful” and “not successful”. No significant effect could be detected (P = 0.497).

A statistically significant association between progression and outcome was found ($\varphi ^{\prime}$ = 0.08; P = 0.014). Due to the fact that the $\varphi ^{\prime}$ value is below the threshold of < 0.1 (which indicates the margin at which a meaningful correlation can be declared) the statement of a significant association between the two variables becomes obsolete. No significant association between the progression and the recovery time could be detected (P = 0.218) (Table 3).

**Table 3:** Outcome of 1479 dogs with thoracolumbar intervertebral disc extrusion distributed according to the progression of clinical signs

Progression	Number of cases	Number of cases with successful outcome
Acute	473	433 (91.5%)
Subacute	489	447 (91.4%)
Stable	517	448 (86.7%)
Total	1479	1328

### Time to surgery

In the bivariate analysis, no statistical association between the time of onset of clinical signs until surgery and the overall outcome was found (P = 0.06), but in a multivariable regression model, a significant correlation was detected (P = 0.027). A significant association between the time of onset of clinical signs until surgery on the time of recovery was evident ($\varphi^{\prime}$ = 0.14; P = 0.003), but this statement could not be confirmed in a multivariable regression model (Table 4).

**Table 4:** Outcome in 1479 dogs with thoracolumbar intervertebral disc extrusion distributed according to the duration of clinical signs until surgery

Duration	Number of cases	Number of cases with successful outcome
Same day	354	326 (92.1%)
1–2 days	453	412 (90.9%)
3–5 days	255	229 (89.8%)
> 5 days	417	361 (86.6%)
Total	1479	1328

### Clinical outcome

There was no statistically significant association between the implementation of physical rehabilitation and the overall outcome (P = 0.990). Although no significant correlation within the patient cohort was detected, differences in grade 5 patients were noticed. The proportion of grade 5 patients that returned to a normal neurologic status increased from 80.0 to 91.4% when physical rehabilitation was implemented. Additionally, the proportion of dogs that had to be euthanized (grade 5) decreased from 20.0 to 2.9% when comparing the group without to the group with physical rehabilitation. However, the hypothesis that dogs of all neurologic grades show a better clinical outcome when physical rehabilitation is performed could not be statistically verified. A significant correlation between the implementation of physical rehabilitation and the recovery time was detected ($\varphi ^{\prime}$ = 0.2; P > 0.001), but this finding could not be verified in a multivariable regression model.

## Discussion

In the present study we addressed specific therapeutic and prognostic questions concerning thoracolumbar intervertebral disc extrusions, which are controversely discussed in the literature to date. Based on a large population of 1501 dogs, we aimed to investigate the impact of the aformentioned factors (duration, progression, grade of severity and physical rehabilitation) on overall outcome and recovery time. Previous studies have shown that factors like the severity of clinical signs prior to surgery including the loss of DPP or the type of surgical treatment have an important impact on the prognosis [1, 3, 5–11].

### Onset of clinical signs

The influence of onset of clinical signs on the overall outcome is a frequently discussed factor. In our study, we could not detect a significant association between acute onset of clinical signs and a worse clinical outcome. This result was verified in a multivariable regression model. Our findings are in accordance with other studies, which have not been able to confirm this association either [2, 10]. Therefore, it may be inappropriate to communicate a worse prognosis due to an acute onset of clinical signs [1]. However, Scott [10] found that a peracute onset of clinical signs is associated with a worse clinical outcome, supposedly caused by a more severe impact on and damage to the spinal cord [15, 16]. The findings of the studies so far vary greatly and due to the different study settings and patient populations it is difficult to reliably compare the studies. Therefore, further prospective studies will be necessary to conclude on this variable.

### Progression

To the authors knowledge, there is no randomized prospective study in veterinary medicine nor in human medicine that focused on the progression of clinical signs as a predictive factor on outcome, as it may appear unethical to postpone surgery or treatment when the clinical signs worsen over time. In the present study we expected to detect an association between an acute deterioration and a worse clinical outcome, but this association could not be established. Although in 32.0% of the patients an acute worsening of the neurologic status was seen, it did not lead to significantly worse clinical outcomes. This might be explained by the fact, that most of patients had intact deep pain perception, which is commonly associated with a good prognosis and outcome despite worsening of their neurological state. Deterioration of the neurologic status usually took place within the first 72 h after onset of clinical signs. Furthermore, no significant statistical association between the variables “progression” and “recovery time” could be found.

### Duration until surgery

As intervertebral disc extrusions are mostly seen as neurologic emergencies, an early surgical intervention is often recommended to achieve a better clinical outcome [10]. In our multivariable regression model, we identified a statistically significant association between the timespan between the onset of clinical signs and surgery and the overall outcome. The observation that outcome is not associated with duration of paraplegia prior to surgery was stated by Olby et al. [1] and is in accordance with results of other studies [10, 17]. In contrast to our findings, Ferreira et al. [2] demonstrated that the duration of clinical signs until surgery did not affect outcome, but that it did affect the recovery time. The authors observed that the time to regain ambulation was prolonged in dogs that had shown clinical signs for more than 6 days. However, the cohort of dogs included in this study was significantly smaller compared to the present study. A recent study evaluated the risk of non-ambulatory dogs to lose DPP if their surgery was delayed [18]. The patients were grouped into either having surgery performed between the day of admission (day 0) and the following morning (day 1), or at a later time. They concluded that immediate surgery might significantly improve the prognosis for some animals by preventing subsequent injury of the spinal cord following the intervertebral disc extrusion. Additionally, the authors postulated that an increased risk of complications might be present for decompressive spine surgeries performed out-of-hours. In accordance to these findings and relying on our observations, we therefore recommend an early surgical intervention in dogs with acute thoracolumbar intervertebral disc extrusion.

### Severity

We were not able to establish a significant association between neurologic grade and outcome. Therefore, our assumption that dogs with severe neurologic grades tend to have worse clinical outcomes could not be verified. This finding was supported by the result of our crosstable, focusing on the assumption that paraplegic dogs with loss of DPP tend to have worse clinical outcomes. No significant correlation could be detected (P = 0.497). In our study, the overall prognosis for dogs without DPP to achieve functional recovery was fair (87.0%) when compared to findings of other studies. The previously reported percentage of patients without DPP achieving functional recovery varies between 38 and 86% [1, 2, 10, 18, 19]. Our results, however, must be interpreted cautiously since only a relatively low number of dogs with no DPP (n = 54) were included in the present study. The percentage of unsuccessful outcomes was slightly lower than what has been reported in previous studies (14.7%) [4].

### Recovery time

The recovery time has been subject to many studies and is one of the key questions of owners, especially in cases where intensive care is required at home. We observed that during the first 4 weeks 72.0% of all patients of this study regained a normal locomotory status. The longest recovery time was seen among patients with no DPP. A statistically significant correlation between neurologic grade and recovery time was detected. This observation is in accordance with the findings of a recently published study, that detected a significant correlation between the grade of severity and the time of recovery (P < 0.001) [20]. Furthermore, in a study by Olby et al. [1] the mean time until paraplegic dogs regained ambulation was 7.5 weeks. A total of 62% recovered ambulation within the first 4 weeks after surgery, which demonstrated a slightly lower percentage than that of our population. The presence of post-surgical voluntary movement has been identified as an important predictive factor by several authors [1, 2, 12, 21]. Based on our results, we recommend that owners of dogs having severe neurologic deficits should be elaborately informed about the possibility of a prolonged recovery time, but further prospective investigations will be required to refine the value of this statement.

### Physical rehabilitation

Physical rehabilitation is a commonly recommended procedure for dogs recovering from intervertebral disc extrusion. Unfortunately, so far the numbers of studies focusing on its effect on clinical outcome or recovery time in dogs with intervertebral disc extrusion are sparse. In our retrospective study, 46.7% of the population underwent physical rehabilitation. Our hypothesis that dogs of all neurologic grades have a better clinical outcome when physical rehabilitation is performed could not be verified statistically. This is in accordance with a randomized clinical trial in 2017 that failed to identify an effect of physical rehabilitation on outcome and a study from 2015, in which physical rehabilitation showed neither a significant influence on outcome nor on the recovery time [22, 23]. Conversely, Ruddle et al. [5] observed a significant difference in time and ability to regain ambulation for dogs that had underwent physical rehabilitation postoperatively in comparison to dogs without this adjunctive treatment. Although we were not able to identify a statistically significant positive effect of physical rehabilitation on outcome, an effect was noted among the 54 patients that were paraplegic with loss of DPP prior to surgery. The percentage of dogs with loss of DPP that had to be euthanised due to a poor neurologic condition decreased from 20.0 to 2.9%, when physical rehabilitation was performed. We assume that patients with severe spinal cord injuries might indeed benefit from physical rehabilitation. In accordance with our observations, another retrospective study which focused on paraplegic dogs without DPP reported a positive effect of physical rehabilitation [24]. Retrospective studies from 2015 and 2017 also focused on the effect of physical rehabilitation on outcome and recovery times; both suggested a benefit of this treatment [25, 26]. A recent study investigated the effect of basic versus intensive postoperative rehabilitation programs on the locomotory recovery of dogs with acute thoracolumbar intervertebral disc herniation [27]. They concluded that an early postsurgical physical rehabilitation was safe, but that it had no beneficial effect neither on the rate of recovery nor on outcome. This blinded and prospective clinical trial excluded dogs that had lost deep pain perception, although the authors assumed that especially dogs with no DPP could benefit from physical rehabilitation considerably. A systematic review of recent studies in human medicine aimed to assess the effect of physical therapy (manual therapy and traction) and its possible influence on the physiology of the lumbar intervertebral disc extrusion [28]. Due to the limited number of reports, the authors were not able to find a single randomized collected trial investigation that focused on this question. The authors concluded that fluid flow, as one of the mechanisms of disc nutrition, can be positively supported by physical rehabilitation. However, they were not able to ascertain whether physical rehabilitation (manual therapy and traction) also has a beneficial effect on pain and disability in patients and therefore stated that further randomized controlled trials were required. Further veterinary studies are also warranted, as the studies on the effect of physical rehabilitation on the outcome of intervertebral disc extrusion so far are inconclusive. Although no statistically significant association could be detected, the authors in alignment with the current literature, suggest that patients might benefit from a postoperative physical rehabilitation program. Furthermore, based on the observation that in dogs without DPP in which physical rehabilitation was implemented, the percentage of cases that had to be euthanised was considerably lower than in those which did not receive physical rehabilitation, owners should be encouraged to try this adjunctive treatment.

Limitations of our study include the retrospective and multicentric nature. This leads to an inherent variability for most of the investigated parameters (for example neurosurgical expertise and intensity of physical rehabilitation).

There is a difficulty in interpreting the underlying biological reason for the association between outcome and physical rehabilitation. Due to the retrospective nature, we are unable to clarify whether physical rehabilitation improves the outcome, or if animals with a worse response to surgery are less likely to undergo extensive rehabilitation. In order to solve this question, further randomized controlled clinical trials will be needed.

In the rare cases in which information concerning outcome and recovery time were lacking, owners had to be interviewed. The assessment by the owners (n = 20) must be interpreted with caution, as it is based on their subjective impression. Furthermore, the treatment and physical rehabilitation protocols varied between institutions. This limitation creates several biases that prohibit the definition of standardized treatment protocols and their effect on outcome and recovery time.

## Conclusions

The results of this study support the recommendation that an early surgical intervention in dogs with acute thoracolumbar disc extrusion is warranted. The neurological grade prior to surgery is significantly related to the recovery time, but even severe cases showed relatively short recovery times. Therefore, it is not advisable to conclude that every patient with more severe neurologic deficits will automatically have a prolonged recovery. Statistically, the beneficial effect of physical rehabilitation could not be proven, although differences in outcome in paraplegic dogs without DPP could be seen. These findings are not finally clarified and further studies are warranted to statistically confirm this finding. As no negative effect of physical rehabilitation on outcome or recovery time could be observed, we would still recommend this postsurgical adjunctive treatment. The analysis of the association between individual variables led to several significant results. When the impact of multiple variables on outcome and recovery time was analysed, it was determinated that they appear to influence one another. Therefore, prognostic statements are further complicated and should be made with caution.

## Data Availability

The data and material used and analysed during the current study are available from the corresponding author on reasonable request.
